# Type 2 Diabetes Mellitus Is Associated with *Strongyloides stercoralis* Treatment Failure in Australian Aboriginals

**DOI:** 10.1371/journal.pntd.0003976

**Published:** 2015-08-21

**Authors:** Russell Hays, Adrian Esterman, Robyn McDermott

**Affiliations:** 1 Kimberley Aboriginal Medical Council, Broome, Western Australia, Australia; 2 James Cook University, Cairns Campus, Smithfield, Queensland, Australia; 3 Centre for Research Excellence in Chronic Disease Prevention, The Cairns Institute, James Cook University, Cairns Campus, Smithfield, Queensland, Australia; 4 Sansom Institute of Health Service Research and School of Nursing and Midwifery, University of South Australia City East Campus, Adelaide, South Australia, Australia; 5 Centre for Chronic Disease Prevention, Australian Institute of Tropical Health and Medicine, College of Public Health, Medical and Veterinary Sciences, James Cook University, Cairns, Queensland, Australia; 6 School of Population Health, University of South Australia, Adelaide, South Australia, Australia; The George Washington University, UNITED STATES

## Abstract

**Objective:**

To explore the efficacy of ivermectin in the treatment of serologically diagnosed cases of *Strongyloides stercoralis* (*S*. *stercoralis*) infection in an Aboriginal community and to describe factors that may influence the outcome of treatment.

**Methods:**

Longitudinal study of a group of 92 individuals with serologically diagnosed *S*. *stercoralis* treated with ivermectin and followed up over a period of approximately 6 months. Main outcomes were serological titers pre and post treatment, diabetic status, and duration of follow up.

**Findings:**

Treatment success was achieved in 62% to 79% of cases dependent on the methods employed for the diagnosis of infection and assessment of treatment outcome. Type 2 Diabetes Mellitus (T2DM) was found to be significantly associated with treatment failure in this group for two of the three methods employed.

**Interpretation:**

Ivermectin has been confirmed as an effective treatment for *S stercoralis* infection in this setting. T2DM appears to be an independent risk factor for treatment failure in this population, and plausible mechanisms to explain this observation are presented.

## Author Summary

In this study we examine the treatment of the intestinal worm infection *Strongyloides stercoralis*, a soil transmitted helminth that is common in the developing world and in the Aboriginal communities of northern Australia. Oral ivermectin is generally accepted as the treatment of choice for this condition. We screened an Aboriginal community for Strongyloides infection over the course of 2 years and measured the outcome of treatment in the 92 cases we found. This study measures the success or otherwise of treatment, and looks at whether there are any factors that could influence the rate of treatment failure. The results suggest that ivermectin is an effective treatment for Strongyloides in this setting, and that pre-existing Type 2 Diabetes Mellitus is a risk factor for treatment failure, an observation of great interest in Aboriginal communities where the prevalence of both conditions is very high. We discuss some possible mechanisms to help explain this previously unknown relationship.

## Introduction

*S*. *stercoralis* is a soil transmitted helminth infection and as such is a neglected tropical disease. It is thought to have a worldwide prevalence of at least 100 million people, and is endemic in regions where people are in contact with fecal material or contaminated soil. The majority of infections in the inhabitants of endemic regions are thought to be asymptomatic, but it can produce a variety of gastrointestinal and skin symptoms. Unusually, *S*. *stercoralis* has the capacity for an auto-infective cycle, and in the presence of host immunosuppression hyper-infection syndrome may develop, resulting in severe morbidity and even death.[1]

In the past, the absence of a gold standard for the diagnosis of this infection, uncertainty as to the most effective treatment, and difficulty in establishing whether a cure has been achieved, has posed problems for research into this condition.[2]

Traditional microbiological methods for detecting strongyloides infection, including concentration techniques for fecal examination such as the Baermann and Harada-Mori techniques, and agar plate culture, require the use of repeated, fresh fecal samples, and have been troubled by low sensitivity. [2] This is particularly so when assessing patients for cure, as larval output can be low and intermittent leading both to under-estimates of the prevalence of the infection, and over- estimates of the effectiveness of treatment.[3].

In recent years serological methods for the detection of strongyloides infection have been developed and advocated as being suitable for both clinical assessment and epidemiological surveys. Furthermore, there is increasing evidence for the use of serological tests to follow up treatment and assess cure.[4]

Various medications have been employed for the treatment of strongyloides infection including albendazole, thiobendazole and ivermectin. It is now generally accepted that ivermectin is the most effective treatment and should be the treatment of choice where available, although questions still remain regarding the best dosing schedule. [3]

Similarly, the best means of follow up after treatment and the appropriate time frame in which to do this has yet to be established. Follow up of ELISA serology has employed either a fall from a positive titer to a negative (treatment success), or a proportional fall in titer such that the ratio of post to pretreatment titers is less than 0.6(treatment effectiveness) [4] The lengthy time frame required for the follow up of cases has often led to high rates of patients lost to follow up.[5].

Strongyloides infection is extremely common in Indigenous communities of the tropical regions of northern Australia with a prevalence of 41% in some settings.[6,7] Despite increasing access to serological tests and adequate treatment, screening and treatment programs remain intermittent, and little is known about the efficacy of ivermectin under Australian conditions, with a recent review noting the paucity of adequate follow up studies following treatment. [5]

In this study we report on the treatment and follow up of 92 serologically diagnosed cases of *S*. *stercoralis* infection in a remote Aboriginal community, and the effect of a variety of epidemiological factors and co-morbidities on the outcome of treatment.

## Materials and Methods

### Study setting

This study was conducted in three isolated indigenous communities located on the edge of the Tanami desert in the Kimberley region of Western Australia. The communities are inter-related and consist of a mobile population of approximately 1200 people. A medical clinic in each community provides primary care and emergency services, and the nearest hospital and laboratory services are some 800km away in the town of Broome.

### Patients

Prior to this study the approach to testing and treating for helminth infection had been intermittent and largely empirical. Patients suspected of having a worm infection were generally given a three-day course of albendazole 400mg without any laboratory testing, and very little ivermectin had been used. In April 2012 it was decided to adopt the best practice guidelines of the Australian Strongyloides working group [8] and patients attending the clinic were subsequently offered testing and treatment on an opportunistic basis for *S*. *stercoralis* infection. As the presumed prevalence of *S*. *stercoralis* in the region was about 37% [7], all Indigenous patients resident in the study communities were considered to be at risk of infection.

Data were extracted including the age, sex, date of testing, *S*. *stercoralis* ELISA titer, haemoglobin, total eosinophil count, percentage eosinophilia, height, weight, calculated BMI, diabetic status and HbA1C triglyceride level, HDL and total cholesterol. (S1 Dataset)

Eosinophilia was defined as a total eosinophil count of 0.50 x10^9^/L or greater.

T2DM was defined in this group as an HbA1C reading of 6.5% or greater, or a random blood glucose of more than 11.1 mmol/l, or a fasting blood glucose of more than 7.0 mmol/l, either at the time of testing, or in the past in patients already receiving treatment for diabetes.

In addition, data were recorded regarding the past treatment, if any, with anthelmintic drugs. Patients were excluded from the study if they had received past treatment with ivermectin in the absence of serological testing.

The patient group in this study constituted part of that utilized in an earlier published study. [9]

### Serological testing

*S*. *stercoralis* ELISA testing was performed by Pathwest Laboratory in Perth Western Australia, using the commercial Strongyloides IgG ELISA (DRG laboratory). The reference values for this test were established in a low prevalence (<1/10000) population, with > 0.40 units of absorption held to be positive, < 0.20 units negative, and values in between considered “equivocal”. As this study was being conducted in a presumed high prevalence setting, and in the interests of avoiding false negative results, a modified range was employed. Values greater than or equal to 0.30 units were considered positive and treated. All values less than 0.30 units were considered equivocal and were re-tested after a period of 6 months.

Clinical treatment success was defined as a fall in titer to < 0.30 units. Only patients who failed to achieve this were, where possible, retreated with ivermectin.

### Anthelminthic treatments

Patients identified as positive for *S*. *stercoralis* under these criteria were treated with two doses of ivermectin 0.2 mg/kg given two weeks apart. All patients, whether positive or equivocal, were recalled for re-testing after a period of 6 months. Although all patients were recalled after 6 months, difficulties in locating subjects for follow up resulted in a wide range (83 to 498 days, median 214 days) of follow up periods. (S2 Dataset).

### Ethics statement

The protocol for this study was approved by the Kimberley Aboriginal Health Planning Forum. As no tests, treatments or interventions were undertaken apart from those required for the accepted Australian best practice [8] for the diagnosis and treatment of strongyloides infection, and all data was de-identified before storage, verbal consent was considered adequate. Literacy levels in written English are low in the communities studied. All subjects were 21 years of age or older. Verbal consent was obtained at the time of initial testing and stored electronically. This approach was endorsed, and subsequent ethical approval for the study was obtained, through the Western Australian Aboriginal Health Ethics Committee (HREC Reference 515).

### Statistical analysis

As the decision to treat at a level of 0.30 units was based only on clinical grounds, and because the best means for determining treatment failure remains uncertain, the data obtained were analyzed in three different ways.

The first definition of treatment failure was an initial titer of ≥0.30 units followed by a subsequent post-treatment reading of ≥0.30 units. In a second analysis failure to achieve treatment “effectiveness”, that is a titer ratio ≥0.60 post-treatment compared to pre-treatment, was the measure of treatment failure. Lastly, the analysis was repeated using the conventional ≥0.40 units as the cut-off point for positivity, and ≥0.40 units post treatment as the measure of treatment failure.

Descriptive statistics are provided with means, median, percentages and their 95% confidence intervals. Bootstrapping was used to obtain the confidence intervals for medians, whereas exact binomial confidence intervals are provided for percentages. Comparisons between those with and without diabetes were tested using independent samples t-tests, Mann-Whitney U-tests and chi-squared tests for normally distributed continuous variables, skewed continuous variables and proportions respectively. Log binomial generalized linear models were used to assess the association between diabetic status and treatment failure after adjusting for age, sex, initial titer, follow up period and eosinophilia, and HbA1c (%) as a predictor of treatment failure. All analyses were undertaken using the Stata 13 statistical package.

## Results

The characteristics of the 259 patients in this study, and the follow up for the 92 positive cases are summarized in Tables 1 and 2. Follow up serology was performed in 87 of the 92 patients treated (94.6%). Of the 5 patients not retested, one was diabetic and 4 were non-diabetic. One patient had deceased, one was in the prison system and 3 were lost to follow up.

**Table 1:** Clinical characteristics of the study participants (N = 259).

Variable	N	Mean, Median or %	95% CI
Age (years)	259	43.4	41.7–45.1
Male	106	40.9%	35.1–47.1
Weight (Kg)	253	81.6	78.9–84.4
BMI (kg/m2)	245	29.7	28.7–30.6
Hb (g/l)	249	133.8	131.7–135.8
HbA1c %	220	6.8^a^	6.5–7.1
SBP (mmHg)	259	126.8	124.5–129.1
DBP (mmHg)	259	79.7	78.3–81.1
Cholesterol (mmol/l)	228	4.6	4.4–4.7
HDL (mmol/l)	227	0.90	0.87–0.93
Triglycerides (mmol/l)	227	2.1^a^	1.9–2.3
Diabetes	131	50.6%	44.5–56.7
Eosinophil count	237	0.43^a^	0.38–0.48
Eosinophil %	236	5.55^a^	4.71–6.39
E (ELISA) titre	259	0.15^a^	0.12–0.18
% E-titre ≥ 0.3	92	35.5	29.7–41.7

^a^ Median

**Table 2:** Characteristics and outcomes for positive cases by diabetes status with 95% CI.

	No diabetes (N = 60)	Type 2 Diabetes (N = 32)	Sig.^a^
Age (years) Mean	41.2 (36.9–45.6)	46.8 (42.7–50.9)	0.098
Male (%)	33.3 (22.0–47.0)	50.0 932.0–68.0)	0.119
Weight (Kg) Mean	74.2 (68.6–79.7)	89.4 (68.6–79.7)	0.005
BMI (kg/m^2^) Mean	27.8 (25.8–29.8)	31.8 (28.3–35.4)	0.031
Hb (g/l) Mean	130.9 (126.1–135.7)	133.4 (126.7–140.1)	0.521
HbA1c % Median	5.9 (5.8–6.0)	8.2 (7.2–9.2)	<0.001
SBP (mmHg) Mean	124.0 (119.5–128.6)	134.5 (126.7–142.4)	0.014
DBP (mmHg) Mean	77.3 (74.5–80.0)	83.1 (79.3–86.8)	0.014
Follow up period (Days) Median	215.5 (198.2–232.8)	213.5 (185.4–241.6)	0.921
Initial serology (Titer) Median	0.57 (0.37–0.77)	0.87 (0.68–1.05)	0.244
Days between 1^st^ and 2nd dose	19.5 (15.6–23.4)	16.0 (12.1–19.9)	0.091
ELISA			
Post-treatment ≥0.30 (%)	31.7 (20.3–45.0)	50.0 (31.9–68.1)	0.085
Post-treatment ratio ≥0.60	25.0 (14.4–38.4)	29.0 (14.2–48.0)	0.683
Pre-treatment >0.40 Post ≥0.40	16.7 (8.3–28.5)	28.1 (13.7–46.7)	0.196
Eosinophilia (%)	54.5 (40.6–68.0)	71.9 (53.3–86.3)	0.789

^a^Independent samples t-tests for continuous variables, Chi-squared tests for categorical variables, Mann-Whitney U-test for skewed continuous variables

**Fig 1:**
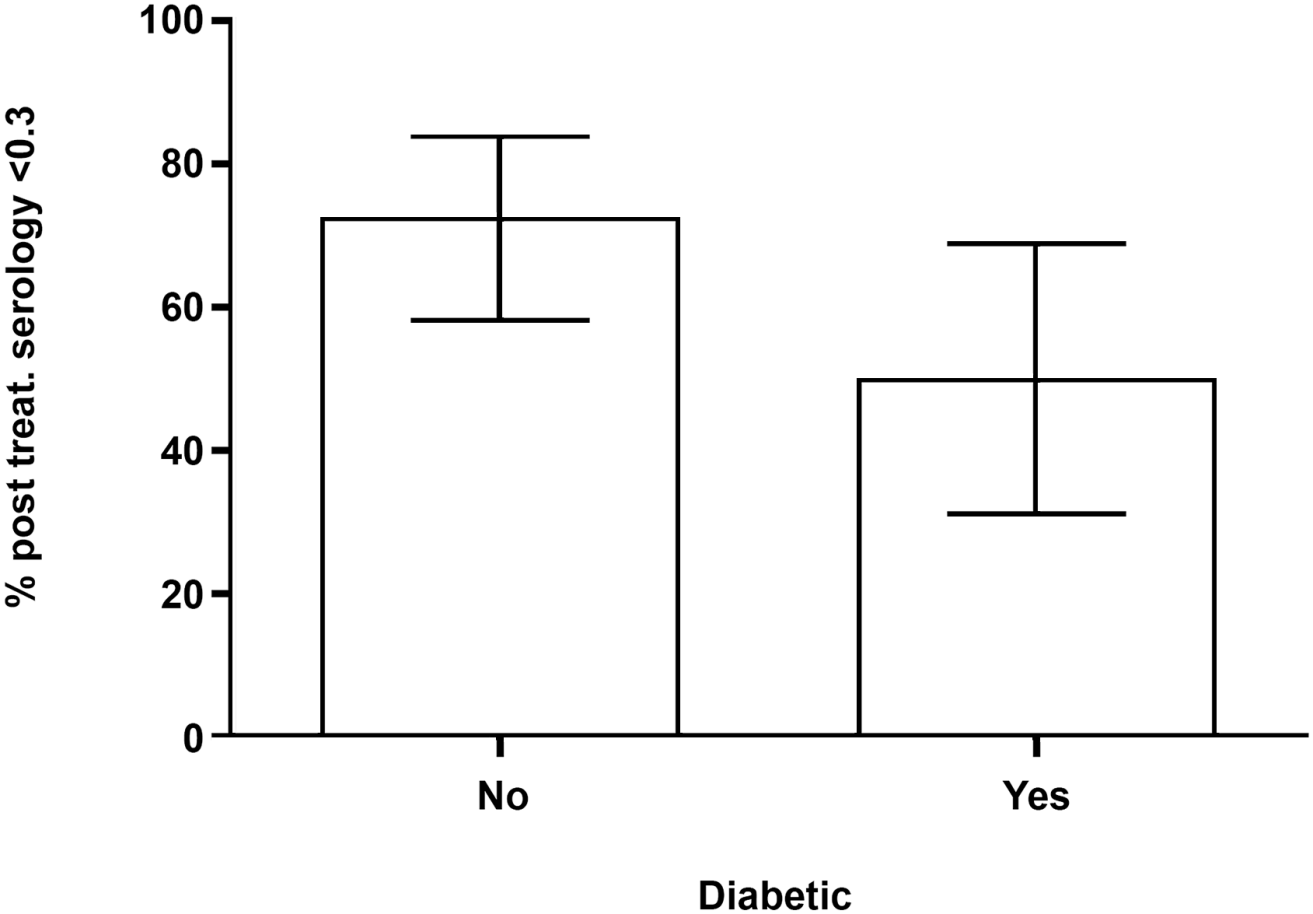
Percentage of diabetic and non-diabetic patients achieving a post-treatment tire of <0.30, with 95% confidence intervals.

Treatment failure as defined by a post-treatment titer of ≥0.30 was seen in 38% of patients. Using the ratio of post-treatment to pre-treatment titer of ≥0.60, 26% of patients had treatment failure. If only those with an initial titer of ≥0.40 and a subsequent reading of ≥0.40 are defined as a failure, then the treatment failure rate was 21%. The percentage of diabetic and non-diabetic patients achieving a post treatment titer of < 0.30 is shown graphically. (Fig 1).

T2DM was predictive of treatment failure after adjustment for age, sex, initial titer, follow up period, treatment interval and eosinophilia, for both cut off points of 0.30 units (p = 0.025) and 0.40 units (p = 0.006). It was not predictive of failure to achieve treatment “effectiveness” (post to pretreatment ratio of >0.60) (Table 3).

**Table 3:** Outcomes for positive cases by diabetes status after adjusting for age, sex, initial titer, follow up period, days between first and second dose and eosinophilia.

	Risk ratio (RR)	95% CI for RR	Sig.^a^
ELISA			
Post-treatment ≥0.30 (%)	1.811	1.076–3.048	0.025
Post-treatment ratio ≥0.60	1.482	0.698–3.147	0.306
Pre-treatment >0.40 Post ≥0.40	3.848	1.478–10.019	0.006

^a^Based on log binomial generalized linear model.

In addition, follow up of the “equivocal” group (initial titer <0.30, N = 167) over an average of 220 days revealed only 3 new cases in this period (1.Female age 53, pre titer 0.19 –post 0.30, 2. Female 43, pre 0.15-post 0.43, 3. Female 30, pre 0.18-post 0.30) suggesting that treatment failures were unlikely to represent incidences of re-infection.

Analysis was performed to determine whether HbA1C, measured as a percentage, was predictive of treatment failure (defined as post treatment titer ≥ 0.30) in the diabetic group and revealed only a weak positive association (RR 1.085 95% CI 0.963–1.222 p = 0.179).

## Discussion

This study confirms the efficacy of ivermectin treatment for strongyloides infection in the setting of an Australian Aboriginal community, although the overall failure rate for treatment is higher than in some previous studies.[10] The use of serology rather than direct microbiological examination for follow up, may in part explain this apparent lower efficacy, as it has been noted that the low sensitivity of microbiological tests tend to overestimate the efficacy of treatment. [3] [11]

Reliance upon serology alone for diagnosis, rather than traditional microbiological techniques could be perceived as a potential weakness of this study, as it is likely that microscopy alone is capable of delivering a specificity of 100%. There is increasing evidence however that serology is adequate for diagnosis, sero-surveys and follow up of treatment in this condition.[4], and serology may be superior to microscopy in assessing response to treatment given the very low sensitivity of microbiological techniques.

The high proportion of patients with T2DM in this study (51%) could also be a contributing factor, given that T2DM is here demonstrated to be a risk factor for treatment failure. The low rate of sero-conversion in “equivocal” patients suggests a low incidence of new infections in the community, and therefore that the apparent treatment failures are unlikely to represent subsequent reinfections.

Most notably this study finds that pre-existing T2DM is an independent risk factor for treatment failure when adjusted for age sex, BMI, initial titer, time between treatments, eosinophilia and duration of follow up.

There are several plausible explanations for the effect of T2DM on treatment outcome. Firstly, the possibility exists that drug interactions between T2DM medications and ivermectin, and the pharmacokinetics of ivermectin, may have some impact on treatment failure rates. The most commonly used diabetic medications in this study were gliclazide and metformin. There are no established interactions between gliclazide and ivermectin, and their metabolic pathways suggest that an interaction is unlikely. [12, 13]

However, poor absorption is known to be a problem with oral ivermectin. [9] Hyperinsulinaemia in itself is known to slow gastric emptying [14] and metformin can produce gastric side effects including loose bowel actions. It is therefore plausible that impaired absorption of ivermectin in diabetic patients taking metformin is a factor in the observed association.

It is also feasible that the altered gut biota known to occur in T2DM patients [15]could influence the absorption of ivermectin.

The complex nature of the interaction between helminth infections and T2DM could mean that the nature of the worm infections found in diabetic patients is different. As has been demonstrated elsewhere, helminth infections can in themselves affect glucose metabolism through a process of immunomodulation [16] and data obtained from this community has confirmed a strong negative relationship between *S*. *stercoralis* infection and T2DM [9]

Consequent to this, it has been suggested that the infections detected in T2DM patients were more likely to represent relatively recent infections (contracted after the onset of T2DM) whereas the infections in non-diabetics were more likely to represent chronic, well-adapted infections with a modified Th2 reaction established in the host. [9] It is known that worm numbers are likely to be higher in early infections and to fall to lower numbers in chronic infections. [17] The higher treatment failure rate in patients with T2DM could therefore be due to a higher average worm burden.

Ivermectin exerts its effect by paralyzing the adult worms, leading to subsequent worm death, and has little effect on the worm larvae. It may be that relatively early infections in patients with T2DM involve higher numbers of auto-infective larvae and that the persistence of these larvae, with the subsequent re-establishment of a patent infection, is responsible for the higher treatment failure.

As is well known, T2DM has been implicated in having a negative effect on both the frequency and outcome of various infections in human beings, through a variety of proposed mechanisms [18,19] and it may be that the observed association simply represents another example of this process. Though this effect has not to date been described in helminth infections, case histories have been published describing strongyloides hyper-infection syndrome in patients where the only known risk factor was T2DM, suggesting that diabetes can play some role in impairing the immune response to this infection.[20] The fact that treatment failure was found to be only weakly associated with HbA1C suggests that glycemic control in itself may not be the main or only factor at play.

Some laboratory evidence exists to suggest that the immune response to helminth infections in patients with T2DM may play a role. A recent paper [21] has explored the relationship between the adipokine resistin, and multiple helminth infections. The authors found that human resistin was both up-regulated in the period after helminth infection, and that this increased level of resistin was associated with an enhanced Th1 immune response, increased worm burden and impaired parasite clearance. They conclude that resistin could have significant implications for the outcome of helminth disease in humans and that increased resistin expression could be predictive of impaired immunity to helminths.

This clearly has implications for individuals with T2DM, as resistin levels have been measured in relation to obesity, T2DM and the metabolic syndrome, and are known to be variably increased in these conditions. [22] It may be therefore that the observation that T2DM is a predictor of treatment failure in S Stercoralis infection reflects once again the presence of a complex and intimate relationship between this helminth infection, the human immune response, and metabolic illnesses such as T2DM.

### Conclusion

This study supports the use of ivermectin for the treatment of strongyloides stercoralis infection in an Aboriginal community where the disease is endemic. The association of T2DM with treatment failure in this community suggests that further investigation into the efficacy of ivermectin in this group is warranted. A plausible mechanism to explain this observation involving the adipokine resistin has been described, and further study into the complex relationship between helminth infection, immunity and metabolic disease would be of value.

Regardless of the mechanism responsible, this study suggests that clinicians should be vigilant when treating T2DM patients for strongyloides infection, ensuring an optimal treatment regimen and careful follow up for these individuals.

## Supporting Information

S1 Dataset All patients. Initial data on all participants in the study.
(XLSX)

S2 Dataset Treatment outcome. Outcome of treatment for all positive cases.
(XLSX)

S1 Checklist STROBE. (DOCX)
