# Supplementary material for: Type 2 Diabetes Mellitus Is Associated with Strongyloides stercoralis Treatment Failure in Australian Aboriginals
Source: PLoS Negl Trop Dis. 2015 Aug 21;9(8):e0003976. doi: 10.1371/journal.pntd.0003976 (PMC4546619; doi:10.1371/journal.pntd.0003976)
Supplement: S1 Checklist — (DOCX) [file pntd.0003976.s003.docx]

| STROBE CHECK LIST  1.  **Title and Abstract**  (*a*) Indicate the study’s design with a commonly used term in the title or the abstract (Abstract Page 1 Line 51) |
| --- |
| (*b*) Provide in the abstract an informative and balanced summary of what was done and what was found (Abstract Page Line 51-54, Page 2 Line 2-6) |

2.

| Introduction | | |
| --- | --- | --- |
| Background/rationale | 2 | Explain the scientific background and rationale for the investigation being reported (Page 2 Line 16-48, Page 3 Line 1-10) |
| Objectives | 3 | State specific objectives, including any pre-specified hypotheses (Page 3 Line 11-14 |

3.

| Methods | | |
| --- | --- | --- |
| Study design | 4 | Present key elements of study design early in the paper (Page 3 Line 29-43) |
| Setting | 5 | Describe the setting, locations, and relevant dates, including periods of recruitment, exposure, follow-up, and data collection (Page 3 Line 20-23, Line 32-35, Page 4 Line 27-31) |
| Participants | 6 | (*a*) Give the eligibility criteria, and the sources and methods of selection of participants. Describe methods of follow-up (Page 3 Line 32-38,Page 4 Line 27-29) |
|  |  | (*b*) For matched studies, give matching criteria and number of exposed and unexposed N/A |
| Variables | 7 | Clearly define all outcomes, exposures, predictors, potential confounders, and effect modifiers. Give diagnostic criteria, if applicable (Page 3 Line 40-49, Page 4 Line 1-27) |
| Data sources/ measurement | 8* | For each variable of interest, give sources of data and details of methods of assessment (measurement).(Page 3 Line 40-48, Page 4 Line 10-22) Describe comparability of assessment methods if there is more than one group N/A |
| Bias | 9 | Describe any efforts to address potential sources of bias (Page 4 Line 2-3, Line13-18) |
| Study size | 10 | Explain how the study size was arrived at N/A |
| Quantitative variables | 11 | Explain how quantitative variables were handled in the analyses. If applicable, describe which groupings were chosen and why (Page 4 Line 42-49 Page 5 Line 1-2) |
| Statistical methods | 12 | (*a*) Describe all statistical methods, including those used to control for confounding (Page 5 Line 4-14) |
|  |  | (*b*) Describe any methods used to examine subgroups and interactions (Page 5 Line 4-14) |
|  |  | (*c*) Explain how missing data were addressed N/A |
|  |  | (*d*) If applicable, explain how loss to follow-up was addressed (Page 5 Line 20-24) |
|  |  | (*e*) Describe any sensitivity analyses Page 5 Line 42-49, Page 5 Line 1-2) |

4.

| Results | | |
| --- | --- | --- |
| Participants | 13* | (a) Report numbers of individuals at each stage of study—eg numbers potentially eligible, examined for eligibility, confirmed eligible, included in the study, completing follow-up, and analysed (Page 6 Tables 1, 2, 3) |
|  |  | (b) Give reasons for non-participation at each stage (Page 5 Line 22-24) |
|  |  | (c) Consider use of a flow diagram N/A |
| Descriptive data | 14* | (a) Give characteristics of study participants (eg demographic, clinical, social) and information on exposures and potential confounders (Page 6 Tables 1,2,3) |
|  |  | (b) Indicate number of participants with missing data for each variable of interest (Page 6, Tables 1,2,3) |
|  |  | (c) Summarise follow-up time (eg, average and total amount) (Page 4 line 29-31,Page 6 Table 2) |
| Outcome data | 15* | Report numbers of outcome events or summary measures over time (Page 6, Table 3) |
| Main results | 16 | (*a*) Give unadjusted estimates and, if applicable, confounder-adjusted estimates and their precision (eg, 95% confidence interval). Make clear which confounders were adjusted for and why they were included Page 6 Table 3) |
|  |  | (*b*) Report category boundaries when continuous variables were categorized N/A |
|  |  | (*c*) If relevant, consider translating estimates of relative risk into absolute risk for a meaningful time period N/A |
| Other analyses | 17 | Report other analyses done—eg analyses of subgroups and interactions, and sensitivity analyses (Page 5 Line 30-40) |

5.

| Discussion | | |
| --- | --- | --- |
| Key results | 18 | Summarise key results with reference to study objectives (Page 5 Line 25-33) |
| Limitations | 19 | Discuss limitations of the study, taking into account sources of potential bias or imprecision. Discuss both direction and magnitude of any potential bias (Page 6, Line 21-25, Page 7 Line 1-5)) |
| Interpretation | 20 | Give a cautious overall interpretation of results considering objectives, limitations, multiplicity of analyses, results from similar studies, and other relevant evidence (Page 7 Line 6-8, Page 8 Line 24-28) |
| Generalisability | 21 | Discuss the generalisability (external validity) of the study results ( Page 8 Line 24-34) |
| Other information | | |
| Funding | 22 | Give the source of funding and the role of the funders for the present study and, if applicable, for the original study on which the present article is based (N/A , provided separately) |
